# Surface reduction in lithium- and manganese-rich layered cathodes for lithium ion batteries drives voltage decay†

**DOI:** 10.1039/d2ta04876k

**Published:** 2022-09-28

**Authors:** Bo Wen, Farheen N. Sayed, Wesley M. Dose, Jędrzej K. Morzy, Yeonguk Son, Supreeth Nagendran, Caterina Ducati, Clare P. Grey, Michael F. L. De Volder

**Affiliations:** Department of Engineering, University of Cambridge 17 Charles Babbage Road Cambridge CB3 0FS UK mfld2@cam.ac.uk; Yusuf Hamied Department of Chemistry, University of Cambridge Lensfield Road Cambridge CB2 1EW UK cpg27@cam.ac.uk; Cambridge Graphene Centre, University of Cambridge 9 JJ Thomson Avenue Cambridge CB3 0FA UK; Department of Materials Science and Metallurgy, University of Cambridge 27 Charles Babbage Road Cambridge CB3 0FS UK; The Faraday Institution, Quad One, Harwell Science and Innovation Campus Didcot OX11 0RA UK; Department of Chemical Engineering, Changwon National University Gyeongsangnam-do 51140 Republic of Korea

## Abstract

Li*-* and Mn-rich layered oxides (Li_1.2_Ni_0.2_Mn_0.6_O_2_) are actively pursued as high energy and sustainable alternatives to the current Li-ion battery cathodes that contain Co. However, the severe decay in discharge voltage observed in these cathodes needs to be addressed before they can find commercial applications. A few mechanisms differing in origin have been proposed to explain the voltage fade, which may be caused by differences in material composition, morphology and electrochemical testing protocols. Here, these challenges are addressed by synthesising Li_1.2_Ni_0.2_Mn_0.6_O_2_ using three different hydrothermal and solid-state approaches and studying their degradation using the same cell design and cycling protocols. The voltage fade is found to be similar under the same electrochemical testing protocols, regardless of the synthesis method. X-ray absorption near edge, extended X-ray absorption fine structure spectroscopies, and energy loss spectroscopy in a scanning transmission electron microscope indicate only minor changes in the bulk Mn oxidation state but reveal a much more reduced particle surface upon extended cycling. No spinel phase is seen *via* the bulk structural characterisation methods of synchrotron X-ray diffraction, ^7^Li magic angle spinning solid state nuclear magnetic resonance and Raman spectroscopy. Thus, the voltage fade is believed to largely result from a heavily reduced particle surface. This hypothesis is further confirmed by galvanostatic intermittent titration technique analysis, which indicates that only very small shifts in equilibrium potential take place, in contrast to the overpotential which builds up after cycling. This suggests that a major source of the voltage decay is kinetic in origin, resulting from a heavily reduced particle surface with slow Li transport.

## Abstract 2

Surface reduction leads to voltage decay in extended cycling of Li*-* and Mn-rich layered oxides (Li_1.2_Ni_0.2_Mn_0.6_O_2_).

## Graphical abstract

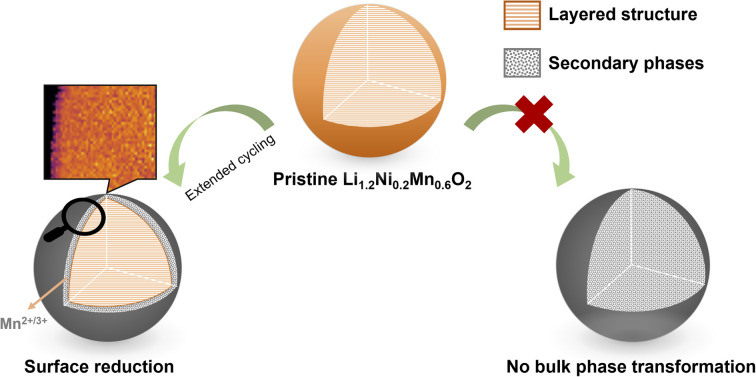

## 1. Introduction

The increasing pace of climate change observed in recent years necessitates faster advancement towards a carbon neutral society, which continuously calls for higher energy density and more environmentally benign cathode materials for Li-ion batteries (LIBs).^1,2^ The classic layered cathode materials, LiNi_*x*_Mn_*y*_Co_*z*_O_2_ (*x* + *y* + *z* = 1) or NMCs, are reaching their theorical limits, yielding gravimetric capacities of ∼140 (NMC111), ∼155 (NMC622) and ∼173 (NMC811) mA h g^−1^ at the active material level upon being charged to 4.2 V *vs.* graphite,^3^ motivating the search for materials that are higher in capacity but contain low or no Co.

Amongst the alternative cathode materials, layered lithium- and manganese-rich oxides have gained considerable interest because of their large gravimetric capacity and energy density (∼200–300 mA h g^−1^ and ∼900 W h kg^−1^, respectively, depending on the composition and electrochemical testing conditions).^4^ A typical composition in this family: Li_1.2_Ni_0.2_Mn_0.6_O_2_ (or Li[Li_0.2_Ni_0.2_Mn_0.6_]O_2_, the elements contained within the square brackets denoting the ions nominally occupying the transition metal layers) is especially promising as the expensive Co is replaced for earth abundant Mn.^5–9^ Layered Li- and Mn-rich oxides are similar to LiCoO_2_ in terms of crystal structure (close-packed layers stacked with *O*3 symmetry), however, the Li stoichiometry is too high to be only accommodated in the Li layers, and the excess Li is stored in the transition metal (TM) layers, often with local or partial ordering of the Li and TMs in honeycomb-like structures.^10^ These materials are associated with characteristic charge–discharge profiles with capacities higher than that simply calculated based on the reversible redox reactions involving Ni. This additional capacity is termed as the anomalous capacity, and its origin has been the subject of much debate over the past two decades.^11–16^ It is now commonly believed that cationic and anionic redox processes collectively contribute: for example, for the specific composition of Li_1.2_Ni_0.2_Mn_0.6_O_2_, Ni^2+^ is oxidised to Ni^4+^ during delithiation to produce at most 126.1 mA h g^−1^ before entering a voltage plateau at ∼4.5 V *vs.* Li, where lattice oxygen is oxidised to accompany further Li^+^ extraction.^16^ The voltage plateau is not seen on the discharge curve and is less pronounced or absent in the subsequent cycles, suggesting that this oxygen loss is associated with significant structural transformations. Prominently layered-to-spinel (or spinel-like) phase transitions and oxygen vacancy densification have been proposed in literatures.^17–20^

Many questions still remain to be answered and the structural transformations that occur during cycling are more complex than sometimes implied. Firstly, the spinel or spinel-like phase is usually observed *via* transmission electron microscopy (TEM), whilst there are also reports that show no formation of secondary phases with X-ray diffraction (XRD).^17,21–24^ Further, online electrochemical mass spectrometry (OEMS) by Teufl, Strehle *et al.* suggests the thickness of the spinel layer is limited to a few nanometres on the particle surface, which inhibits further O_2_ release after the first cycle.^25,26^ Yu *et al.* found that this reconstructed surface layer could significantly affect the kinetic processes and lead to voltage hysteresis and decay.^27^ The impact of electrochemical cycling will also be discussed in the present work (Section 4) with an emphasis on the differences between the surface and the bulk. Secondly, bulk oxygen vacancy densification resulting from bulk intra- and inter-layer TM migration and associated Li elimination cannot explain the only minor capacity degradation over long-term cycling. A recent report points out that bulk densification is as small as ∼15%.^23^ Instead, the authors propose that the majority of the bulk oxygen vacancies persist and do not undergo a densification process. The mechanisms of the anion redox of 3d transition metal based layered oxides are controversial, the discussion of which is beyond the scope of this work, and we refer to Zhang *et al.*, for example, for a recent comprehensive review on the topic.^28^ Instead, this work focuses on the so-called voltage drop, *i.e.* a gradual reduction in nominal discharge voltage during cycling, which limits the commercial viability of these materials.^6^

An important challenge within the current literature is that different synthesis methods, cell designs, electrolytes, the nominal battery performance of Li-rich cathodes and cycling protocols are used by different research groups. Such discrepancies heavily complicate the interpretation of different suggested degradation mechanisms.^29–32^ Thus, it is important to understand overarching degradation patterns that are inherent to Li- and Mn-rich cathodes irrespective of synthesis routes, and under well-controlled characterisation conditions.

Here we report the synthesis of Li_1.2_Ni_0.2_Mn_0.6_O_2_*via* an autoclave hydrothermal (HT), a microwave hydrothermal (MHT) and a solid-state (*i.e.* ball milling, BM used as the acronym hereafter) approach, the products of which all revealed similar morphological, crystallographic and electrochemical features. The representative material, MHT, was taken forward for an in-depth study. Specifically, the structural transformations were scrutinised by synchrotron XRD (SXRD), ^7^Li magic angle spinning solid-state nuclear magnetic resonance (MAS ssNMR) and Raman spectroscopy. The X-ray absorption near-edge structure (XANES) experiments and scanning transmission electron microscope-electron energy loss spectroscopy (STEM-EELS) mapping were carried out to reveal the TM oxidation state changes both globally and locally in the particles. This study suggests that the degradation of the Li_1.2_Ni_0.2_Mn_0.6_O_2_ surface and sub-surfaces is a main contributor to the voltage drop which is plaguing this class of materials. This hypothesis is further confirmed by GITT analysis which indicates that only very small shifts in equilibrium potential are taking place whereas the overpotential is building up after cycling. This kinetic Li transport limitation on the reduced cathode surface is frequently overlooked in the literature and informs future research directions on coating, surface-specific doping and other protection mechanisms.

## 2. Experimental

### 2.1 Li_1.2_Ni_0.2_Mn_0.6_O_2_ synthesis

HT approach: the synthesis method was modified from Zheng *et al.*^31^ In brief, stoichiometric amount of LiCH_3_COOH·2H_2_O (5% in excess), Ni(CH_3_COOH)_2_·4H_2_O and Mn(CH_3_COOH)_2_·4H_2_O (Acros Organics) were added in a beaker together with polyvinylpyrrolidone (Fluka Analytical, average molecular weight 360 000), DI water and ethylene glycol (solvents in 1 : 1 volume ratio, Acros Organics). The mixture was stirred rigorously for 30 min before the uniform solution was transferred to a 110 mL PTFE liner and sealed in an autoclave. It was then kept isothermal at 200 °C for 16 h. The reactants were then cooled, decanted to an alumina crucible and heated on a hot plate set at 300 °C to evaporate solvents until a dense slurry was obtained. The slurry was then subjected to a pre-calcination step in a box furnace (MTI KSL-1100X) in air at 450 °C for 12 h, cooled down naturally, and ground thoroughly with agate pestle and mortar for 30 min. The material was then calcined at 900 °C for 24 h to obtain the final product.

MHT approach: the synthesis method is the same as the HT, except the hydrothermal treatment of the precursors was carried out in a microwave reactor (Anton Paar, Multiwave Pro), in which the precursors were heated to 200 °C in 20 min and held at the same temperature for 3 h.

BM approach: the synthesis method was adopted from Xu *et al.*^33^ In brief, Li_2_CO_3_, Ni(OH)_2_ and MnCO_3_ (Sigma Aldrich) were mixed in a ball mill (Retsch PM100) at 450 rpm for 3 h before being calcined at 950 °C in air for 15 h.

### 2.2 Cell assembly and electrochemical test

The synthesised Li_1.2_Ni_0.2_Mn_0.6_O_2_ was mixed with conductive carbon Super P (Cambridge Energy Solutions) and polyvinylidene fluoride (PVDF) binder (MTI, pre-dissolved in 1-methyl-2-pyrrolidinone, NMP, Sigma Aldrich) in a weight ratio of 80 : 10 : 10 in a Thinky mixer at 2000 rpm. Extra NMP was added intermittently to adjust the viscosity of the slurry. The slurry was cast on aluminium foil with a doctor blade and a linear coater. The cast electrode was pre-dried on a hot plate at 120 °C for 30 min before being transferred into a vacuum oven in which it was dried at 120 °C for 6 h. The resulting cast was punched into 12 mm disks, weighed and re-dried in the vacuum oven at the same temperature overnight. The 2032 coin cells (stainless steel coin cell parts from Cambridge Energy Solution) were then assembled in an Ar filled glovebox (O_2_ and H_2_O < 0.5 ppm), using 55 μL of LP40 electrolyte (1 M LiPF_6_ in ethylene carbonate (EC)/diethyl carbonate (DEC), 1 : 1 volume ratio, Sigma Aldrich), Celgard polymer separators (19 mm in diameter, MTI) and Li metal disc counter electrodes. Crimping pressure was kept constant at 1000 psi for all cells. The cells were rested for 12 h before galvanostatic charge and discharge at a constant current density of 20 mA g^−1^ in a voltage window of 4.8–2 V for 3 times as an electrochemical formation process. The current densities were calculated based on the mass of active material unless otherwise specified. Then, the cells were cycled at C/2 (∼112.0 mA g^−1^) and C/5 (∼44.8 mA g^−1^) in the same voltage window for long-term tests. Thus, the current, 20 mA g^−1^, used in the first three cycles corresponds to a C-rate of approximately C/11 based on a reversible capacity of 224 mA h g^−1^. For galvanostatic intermittent titration (GITT) experiments, the cells were given 1 h pulses at C/10 (∼22.4 mA g^−1^) followed by 12 h rests to achieve a quasi-equilibrium state. For the sample cycled at C/2 for 100 cycles, 2 recovery cycles at C/10 were performed before the GITT experiment. The galvanostatic cycling tests were carried out using Landt CT2001A Battery Testing Systems whereas GITT experiments were done using Biologic BCS 805 Series. All electrochemical tests in this study were carried out in an incubator in which the temperature was kept at 25 °C. Unless otherwise specified, all cells in this study were cycled against a Li metal counter/reference electrode.

### 2.3 Post-mortem sample collection

Cycled cells were de-crimped in the Ar filled glovebox. The electrodes were extracted, rinsed in dimethyl carbonate (DMC, anhydrous, Sigma Aldrich), and dried in dynamic vacuum for 1 h for subsequent characterisation.

### 2.4 Powder/synchrotron XRD (P/SXRD)

PXRD spectra were collected on a Panalytical Empyrean diffractometer (Cu Kα = 1.54056 Å) at room temperature in Bragg–Brentano geometry. The powder samples were packed on a flat circular glass holder and scanned in a 2*θ* range of 5–90°. For the SXRD experiment, materials were scrapped off from the extracted electrodes and packed into 0.5 mm diameter quartz capillaries in the Ar filled glovebox, then sealed with two component epoxy to ensure air-tightness. The SXRD experiments were performed at Beamline I11 of Diamond Light Source, UK, using an X-ray wavelength of 0.82686 Å.

### 2.5 Rietveld refinement

The diffraction patterns are refined using GSAS 2 based on the α-NaFeO_2_ structural model with a space group of *R*3̄*m*. Li, Ni and Mn were placed on the octahedral sites of TM layers whereas Li and Ni were placed on the octahedral sites of Li layers. The parameters concerning background, sample displacement, lattice parameters, crystallite size, micro-strain, site occupancy and oxygen positions were refined. Mn was excluded from the site occupancy refinement due to the large mismatch of Mn ion radii compared to Li. The atomic displacement parameters were assumed to be isotropic and fixed at 0.8 Å for Li and TMs, and 1.1 Å for O. The absorption *μ*·*R* = 1.49 was factored in all SXRD refinements.

### 2.6 ^7^Li MAS ssNMR

Electrode materials were scrapped off from current collectors, rinsed, dried and packed into 1.3 mm rotors in an Ar filled glovebox (O_2_ and H_2_O < 0.1 ppm). The experiments were carried out on a 4.7 T Bruker Avance III spectrometer (200 MHz ^1^H Larmor frequency) at 50 kHz MAS using magic-angle turning phase-adjusted sideband separation (MATPASS) pulse sequence. The excitation pulse was set at 480 ppm and a pulse length (P1 at 90°) of 1.1 μs (∼227 kHz) was used, to help ensure that the excitation frequency covers the whole range of observed peaks (including the diamagnetic peak at 0 ppm and the main peak at approx. 700 ppm). The recycle delay and number of scans were 20 ms and 80 000, respectively, for all samples. The peak of solid Li_2_CO_3_ at 0 ppm was used as a reference for ^7^Li chemical shift.

### 2.7 Raman spectroscopy

Electrodes were sealed between a quartz substrate and a piece of cover glass using two component epoxy in the Ar filled glovebox to ensure air-tightness during measurements. The experiments were carried out using a Renishaw Invia Raman spectrometer with a laser wavelength of 514 nm. The laser output power was set at 10%, and the acquisition time was set to 30 s with 2 accumulations.

### 2.8 X-ray absorption spectroscopy (XAS)

Electrode materials were scraped off current collectors, rinsed with DMC and dried before being mixed with pre-dried carboxymethyl cellulose (CMC) and pressed into pellets in the Ar filled glovebox, which were then sealed in an aluminium pouch after placing in a 3D printed frame. The experiments at Mn and Ni K-edges were carried out at the B18 beamline, Diamond Light Source, UK.

### 2.9 STEM-EELS

Scraped powder from extracted electrodes was dusted onto copper mesh, holey carbon film TEM grids (Agar Scientific) immediately before measurement to reduce exposure to oxygen and moisture (about 1 min of exposure). An FEI Tecnai Osiris microscope with a high brightness X-FEG gun was used at 200 kV accelerating voltage in scanning mode. Images were acquired using a high angle annular dark field detector. Gatan Enfinium 977 spectrometer was used to acquire electron energy loss spectrum images. Dual-EELS mode was used, acquiring the low-loss and core-loss regions of the spectra simultaneously. This allowed for later alignment of the zero-loss peak. Pixel size of about 3 nm and dwell time of 0.1 s were used (at probe current 0.1 nA). The resulting spectrum images were analysed using HyperSpy.^34^ Briefly, the zero-loss peak was aligned to 0 eV, along with shifting the core-loss spectrum for each pixel. Then Mn L-edge was fitted at each pixel with: (i) a power law background, (ii) two Hartree–Slater generalised-oscillator-strength based ionisation edges, (iii) two Gaussian peaks (for the *L*_3_ and *L*_2_ white line peaks), and (iv) their convolution with the low-loss spectrum to account for the sample thickness. Maps of Mn *L*_3_ gaussian peak position were therefore produced. To acquire the line scans, a rectangular region of interest of the spectrum image was positioned across the particle surface and averaged perpendicular to that surface to improve signal to noise ratio. This way, excessive beam damage was avoided, while providing a profile of Mn *L*_3_ peak position change across the surface.

## 3. Results

### 3.1 Synthesis and bulk characterisation

**Fig. 1:**
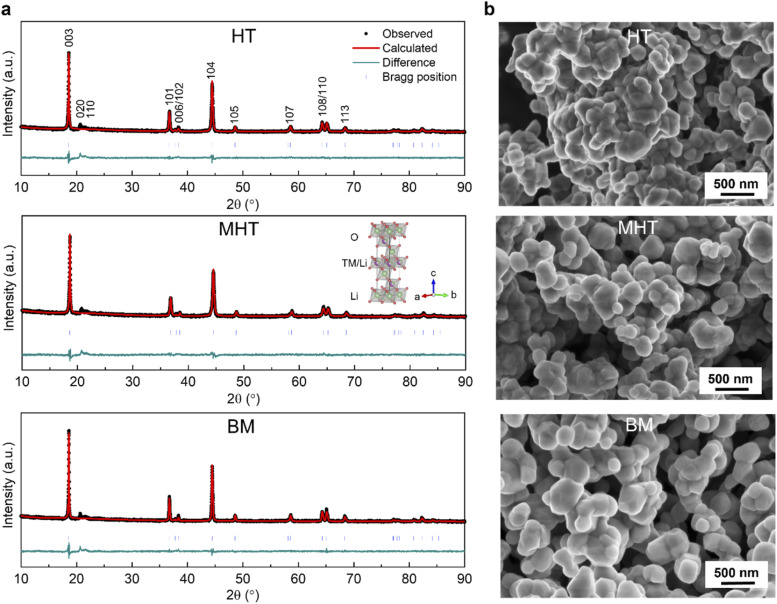
Crystallographic and morphological characterisation of pristine Li_1.2_Ni_0.2_Mn_0.6_O_2_ synthesised *via* different routes. (a) Rietveld refinement of PXRD patterns with a *R*3̄*m* space group for Li_1.2_Ni_0.2_Mn_0.6_O_2_ samples from HT (top), MHT (middle) and BM (bottom). The inset in the middle panel illustrates an unit cell schematic. (b) SEM images showing representative material morphologies for HT (top), MHT (middle) and BM (bottom). The majority of primary particles are 100–500 nm in diameter.

The PXRD patterns of the Li_1.2_Ni_0.2_Mn_0.6_O_2_ synthesised *via* the HT, MHT and BM routes are shown in Fig. 1a. The Rietveld refinements were carried out based on the α-NaFeO_2_ structural model (*R*3̄*m* space group), yielding similar lattice parameters for all three materials as summarised in Table S1†. From a holistic perspective, the diffraction pattern of Li_1.2_Ni_0.2_Mn_0.6_O_2_ resembles that of a traditional stoichiometric (Li : TM = 1 : 1) *O*3-stacked oxide apart from the pronounced superstructural peaks in the range of 20–35° (Cu Kα, *λ* = 1.54056 Å), which result from the honeycomb ordering of the Li and Mn ions within the TM layers and the partial ordering of the honeycomb structure along the *c*-axis.^10,35,36^Fig. 1b shows the representative morphologies of the as-synthesised Li_1.2_Ni_0.2_Mn_0.6_O_2_ samples, with similar primary particle sizes mostly distributed in a range of 100–500 nm.

**Fig. 2:**
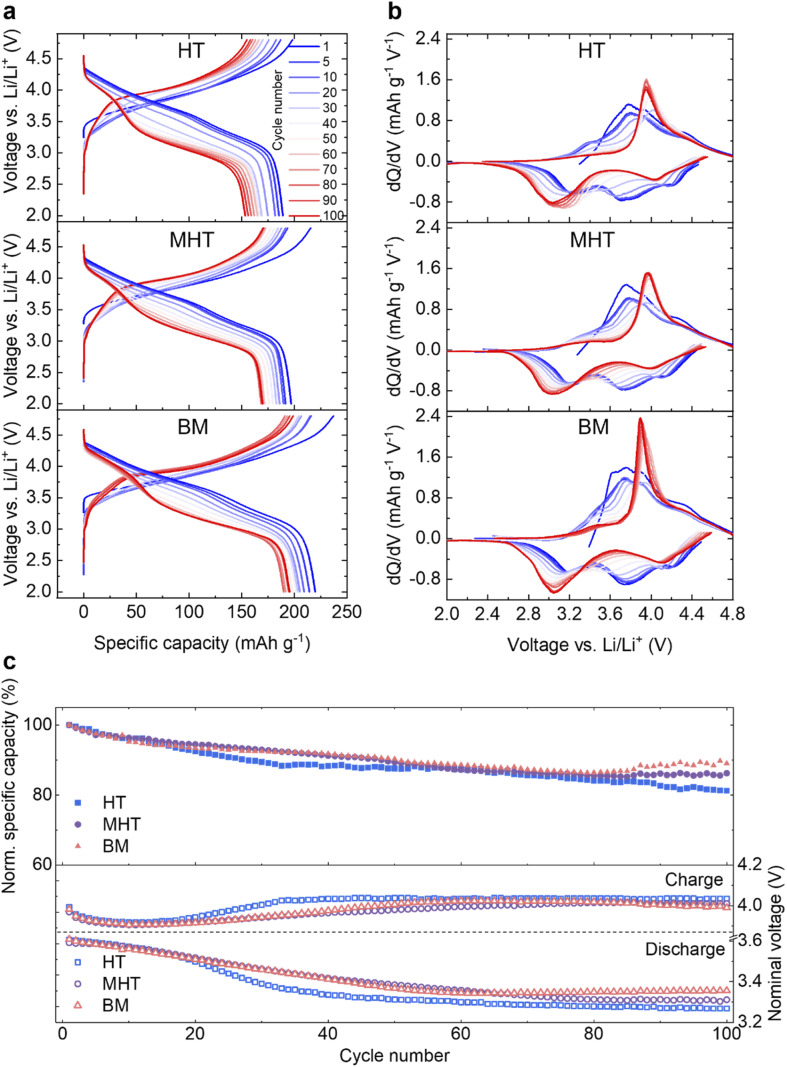
Electrochemical characterisation of Li_1.2_Ni_0.2_Mn_0.6_O_2_ synthesised from different routes. (a) Galvanostatic cycling of Li_1.2_Ni_0.2_Mn_0.6_O_2_ for HT (top), MHT (middle) and BM (bottom) samples at C/5 for 100 cycles in a voltage window of 4.8–2.0 V *vs.* Li. Here, cycle 1 is the first cycle after three formation cycles at 20 mA g^−1^. (b) Evolution of d*Q* d*V*^−1^ plot for 100 cycles derived from (a). (c) Comparison of the normalised specific capacities (top) and nominal charge/discharge voltage (bottom).

Pristine Li_1.2_Ni_0.2_Mn_0.6_O_2_ samples were then electrochemically tested in half-cells against a Li metal counter/reference electrode (see Experimental section). The first cycle reversible specific capacities are 217.3, 227.6 and 242.7 mA h g^−1^ and the initial coulombic efficiencies are 76.3%, 74.6% and 78.2% for HT, MHT and BM samples, respectively (Fig. S1†). These results are in good agreement with materials synthesised *via* other routes in literature, for example, sol–gel and co-precipitation.^22,30,31,37^ The rate performance of the samples are presented in Fig. S2.† During extended cycling, the electrochemical behaviour evolves in a similar way for all three samples over the course of 100 cycles both in terms of capacity degradation and redox potential evolution (Fig. 2a and b). The d*Q* d*V*^−1^ plots of the discharge process, taking the MHT as an example, show that the reduction peak initially at ∼4.15 V gradually shifts to ∼4.03 V; the peak originally located at ∼3.75 V disappears after 100 cycles and the peak at ∼3.21 V shifts to ∼3.04 V, contributing to the voltage decay. The HT, MHT and BM samples retain 81.2%, 86.2% and 89.0% of their initial capacity respectively after 100 cycles (Fig. 2c, top). Deemed as one of the most significant factors determining the gravimetric energy density, the nominal discharge voltage (calculated *via* dividing energy density by specific gravimetric capacity) of all three samples experiences a slow and steady drop during the first 50 cycles, which then flattens out as cycle number accumulates (Fig. 2c, bottom). The nominal charge voltage is shown in the same panel, and increases slowly over cycling, which is in agreement with previous studies.^21,22,27^ As the MHT sample showed intermediate electrochemical performance among the three in comparison, it was chosen for further in-depth studies. The same material cycled at a higher C-rate (C/2, Fig. S3†) also showed a similar degradation pattern, which was used for all studies that follow. Hereafter, for the simplicity of communication, the MHT samples that are pristine, after three formation cycles at 20 mA g^−1^ and after 100 cycles at C/2 are referred to as *Pristine*, *Formed* and *Cycled*, respectively.

**Fig. 3:**
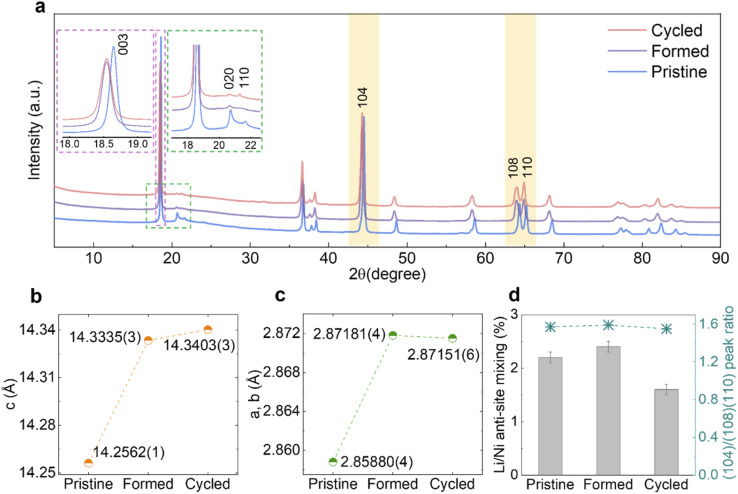
Structural evolution of MHT Li_1.2_Ni_0.2_Mn_0.6_O_2_ over long-term cycling. (a) SXRD patterns of the *Pristine*, *Formed* and *Cycled* samples, with the X-ray wavelength converted to Cu Kα. The inset outlined by the magenta dotted line shows the evolution of the (003) peak, while the inset outlined by the green dotted line shows the evolution of the superstructure peaks. (b and c) Lattice parameters *c* and *a* (=*b*) and their evolution derived from Rietveld refinements. (d) Li/Ni anti-site mixing at different stages and the ratio of the integrated area under the peaks of (104) and (108)/(110) doublet. Error bars are applied to the data in (b), (c) (too small to be visible) and (d).

The samples were characterised by SXRD (*λ* ≈ 0.82686 Å) and Fig. 3a shows the diffraction patterns of *Pristine*, *Formed* and *Cycled* samples in their discharged (fully lithiated) states. The (003) peaks shown in the left inset (magenta) of Fig. 3a, shift to lower 2*θ* angles, which indicates an enlargement of the *c* lattice parameter as quantified by Rietveld refinement (Fig. 3b). At the same time, the *a* and *b* lattice parameters also expand (Fig. 3c), which together translate into a unit cell volume expansion from 100.902(2) Å^3^ (*Pristine*) to 102.375(3) Å^3^ (*Formed*) and eventually to 102.402(4) Å^3^ (*Cycled*). The refined patterns and parameters are shown in Fig. S4a–c and Table S2–S4.†

The asymmetric broadening of superstructural peaks for *Pristine,* shown in the right inset (green) of Fig. 3a, is attributed to honeycomb stacking faults as demonstrated by Bréger *et al.*^10^ As expected, the intensity of the superstructural peaks significantly reduces after the formation cycles. This is probably because at the high state of charge (SOC) reached in the first cycle (SOC_max_ = 79.3% at 4.8 V, assuming a capacity of 378.3 mA h g^−1^), the Li vacancies formed in the TM layers facilitate TM migrations, which in turn could result in disordering of the in-plane honeycomb structures in the TM layers as well as their long-range ordering along the *c*-axis. Interestingly, the rate at which the superstructure peaks fade slows down significantly after the formation cycles and these peaks are still clearly present in the diffraction patterns after 100 cycles. Similarly, the lattice parameters, as well as the unit cell volume, change substantially during formation cycles but only modestly thereafter. To examine the occupancy of TMs in the tetrahedral sites of the Li layers, the ratio of areas under the (104) reflection and (108)/(110) doublet (yellow-shaded) was calculated (Fig. 3d, right axis),^23^ which does not vary noticeably. Furthermore, attempts to refine any site occupancy in the tetrahedral sites of the Li layers did not lead to any improvements in the fits to the patterns. Li/Ni anti-site mixing, indicating the TM migration to the octahedral site of Li layers, was extracted from Rietveld refinement (Fig. 3d, left axis), which does not increase over extended cycling, either. This evidence strongly suggests that no spinel phase formation took place in the bulk of material.

### 3.2 Local structural probes

**Fig. 4:**
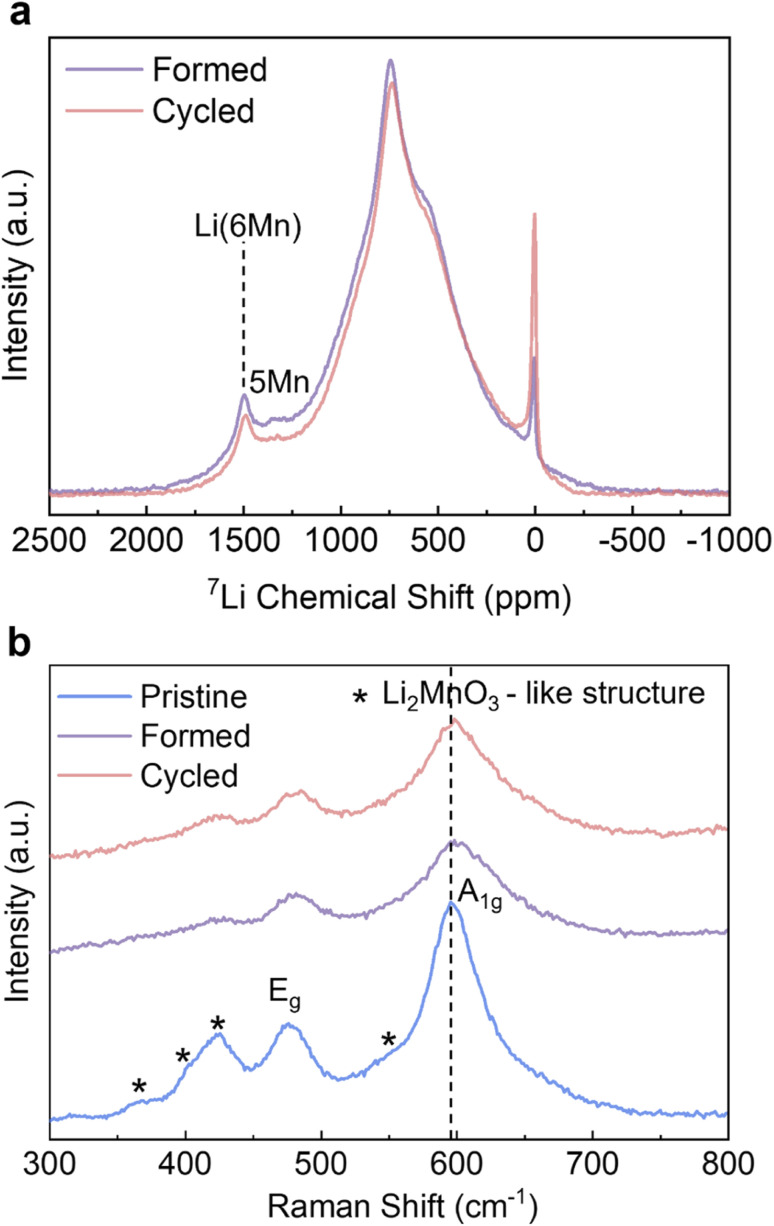
Solid state NMR and Raman analysis. (a) ^7^Li MAS ssNMR MATPASS spectra of the *Formed* and *Cycled* samples and (b) Raman spectra of the *Pristine*, *Formed* and *Cycled* samples. All measurements are taken under an inert gas atmosphere. Pertinent peaks are assigned.

To complement the long-range ordering analysis of Li_1.2_Ni_0.2_Mn_0.6_O_2_ using SXRD, we used ^7^Li MAS ssNMR to probe the Li local environment changes. Two main resonance groups can be identified in the projected sum of the tilted MATPASS spectra (Fig. 4a). The group at 500–800 ppm is assigned to Li in the Li layers, whose large width is ascribed to the presence of a variety of different environments (Ni^2+^, Mn^4+^ and Li^+^) in the first and second coordination shells of Li.^10,38^ The other prominent resonance group, located in the range of 1200–1500 ppm, is assigned to Li in the TM layers. The peak at ∼1500 ppm is attributed to Li^+^ surrounded by 6 Mn^4+^ (Li(OMn)_6_) and the peak at ∼1325 ppm to Li^+^ surrounded by 5 Mn^4+^ and 1 Ni^2+^.^10^ Compared to the spectrum of the *Pristine* sample (shown in Fig. S5†), all the resonances broaden after formation cycles, indicating a loss of ordering.^38^ The spectra were normalised to the weight of active materials loaded in the rotors and number of scans (=80 000 for all samples), hence the integrated area under the peaks correspond to the total Li content in the samples. The area under the peaks reduces by 8.0% (between *Formed* and *Cycled*), which correlates well with the results of galvanostatic cycling where the specific capacity reduces by 10.9%. The intensities of both Li in Li layers and Li in TM layers decrease when comparing the *Formed* against the *Cycled* sample, hence these environments collectively contribute to the capacity degradation. Nevertheless, the TM layers are still able to accommodate Li^+^ after 100 cycles, indicating there is no major bulk TM migration and densification that would eliminate all Li sites in the TM layer, which agrees with the observation of honeycomb superstructure peaks by SXRD after cycling. The diamagnetic ^7^Li peak at 0 ppm becomes more prominent after cycling, which we tentatively attribute to the accumulation of the Li-containing diamagnetic cathode electrolyte interphase (CEI – containing inorganic salts such as LiF and lithium (fluoro)phosphates) formed on the particle surfaces.^38,39^ However, no prominent new local Li environments were detected (such as spinel) despite the high signal-to-noise ratio of the ^7^Li NMR spectra.

*Ex-situ* Raman was carried out to identify the formation of any new phases as it is also highly sensitive to local bonding changes resulting from layered to spinel transitions.^40^ The peaks of the pristine sample at 596 cm^−1^ and 476 cm^−1^ in Fig. 4b can be attributed to *γ*_MO6_ vibration (M = Ni/Mn, *A*_1g_ symmetric stretching) and *δ*_O-M-O_ vibration (*E*_g_ symmetric deformation); whereas the hump at 550 cm^−1^, and the peaks at 424 cm^−1^, 408 cm^−1^ and 360 cm^−1^ are assigned to vibrations originating from Li_2_MnO_3_-like environments with a *C*2/*m* symmetry.^40–42^ The intensities of all peaks decrease and the peaks become broader after the formation cycles, signifying an increase of disorder of Li_1.2_Ni_0.2_Mn_0.6_O_2_. This is particularly pronounced for the peaks attributed to the Li_2_MnO_3_ structure, which agrees well with the decreased intensity of superstructure peaks in the SXRD results. However, after extended cycling the peak shapes and intensities remain the same with very minor shift in peak positions (*e.g.* from 596–599 cm^−1^ for *A*_1g_ peak) compared to *Formed*. The cubic spinel LiMn_2_O_4_ with *Fd*3̄*m* symmetry shows a characteristic *A*_1g_ peak at ∼630 cm^−1^,^40^ which is not observed in the pattern of the *Cycled* sample. Similar to SXRD and ^7^Li ssNMR data, the Raman results also show that the most prominent changes take place during the formation cycles, and the changes substantially slow down thereafter.

### 3.3 Oxidation state changes

**Fig. 5:**
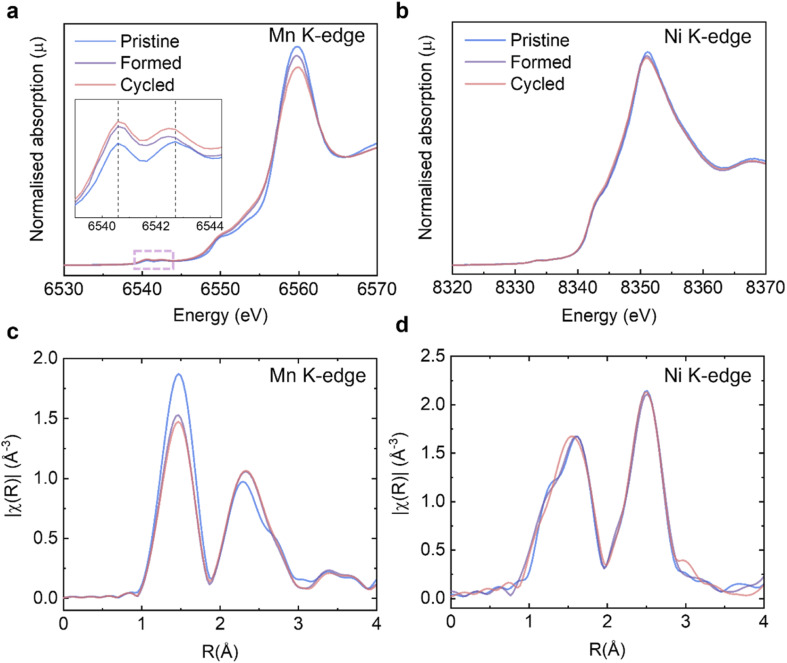
XAS analysis on Li_1.2_Ni_0.2_Mn_0.6_O_2_ structural changes. Evolution of XANES at the (a) Mn; (b) Ni K-edges. Inset in (a) shows an enlargement of the pre-edge features. Evolution of EXAFS at the (c) Mn and (d) Ni K-edges.

On the basis of the long-range and short-range crystallographic information provided by XRD, ssNMR and Raman, we conclude that the spinel phase, if there is any, is present in insufficient quantities in our samples to be captured by these highly phase-sensitive characterisation tools. Another key issue to be explained is the significant discharge voltage fade, also referred to as “droop”, which can be seen in the cycling data (Fig. 2a and S3a†) and the nominal discharge voltage plot (Fig. 2c and S3c†). It is generally believed that the Mn^3+^/Mn^4+^ couple and its lower redox potential (than that involving the Ni^2+^/Ni^4+^ ions) is primarily responsible for the voltage drop.^43^ During the cycling process, some lattice oxygen is released as oxygen gas, or *via* reactions with the electrolyte and hence the oxidation state of a portion of Mn^4+^ must be reduced down to maintain overall charge neutrality. Therefore, it is necessary to (i) quantitatively determine how much Mn^4+^ is reduced as the consequence of galvanostatic cycling and (ii) identify if the surfaces of Li_1.2_Ni_0.2_Mn_0.6_O_2_ are disproportionately impacted as compared to the bulk. The XAS experiments at TM K-edges were carried out on the *Pristine*, *Formed* and *Cycled* samples (Fig. 5a and b, full spectra are presented in Fig. S6†) to assess the overall TM reduction during cycling whereas EELS mapping was used to contrast surface and bulk processes (see further). The XANES at the Mn K-edge shows that the intensity of the pre-edge features (Fig. 5a, inset) increase over cycling, which is attributed to increased distortions in the metal coordination environments.^44^ Such changes are also observed in stoichiometric cathodes such as LiNi_1/3_Mn_1/3_Co_1/3_O_2_.^45^ No noticeable changes take place at the Ni K-edge (Fig. 5b). To determine the Mn valence states quantitatively, four Mn standards (MnO, Mn_3_O_4_, Mn_2_O_3_, and MnO_2_) were measured along with our Li_1.2_Ni_0.2_Mn_0.6_O_2_ to establish an empirical relation between X-ray edge position and oxidation states (Fig. S7a and b†). In this case, the half-height method was applied,^46^ where the edge energy was represented by the energy corresponding to the normalised absorption coefficient *μ* = 0.5 of the normalised XANES spectra. With the knowledge of the edge energy of the standards and their respective oxidation states an empirical linear relationship was established (*R*^2^ = 0.985). From this relationship, the edge energy of the Li_1.2_Ni_0.2_Mn_0.6_O_2_ samples can be converted into the average Mn valence state: for the pristine Li_1.2_Ni_0.2_Mn_0.6_O_2_ it is +3.85, the valence state is then reduced to +3.69 after formation and then to +3.64 after 100 cycles.

The extended X-ray absorption fine structure (EXAFS) at the Mn K-edge (Fig. 5c) shows that the scattering intensity of the first coordination shell reduces after formation whereas that of Ni (Fig. 5d) does not change noticeably, which may suggest O vacancies and/or structural changes (*e.g.* a Jahn–Teller distortion) occur preferentially around Mn.^23^ The trend of scattering intensity decrease, like other structural characterisation results presented in previous sections, slows down significantly after formation.

**Fig. 6:**
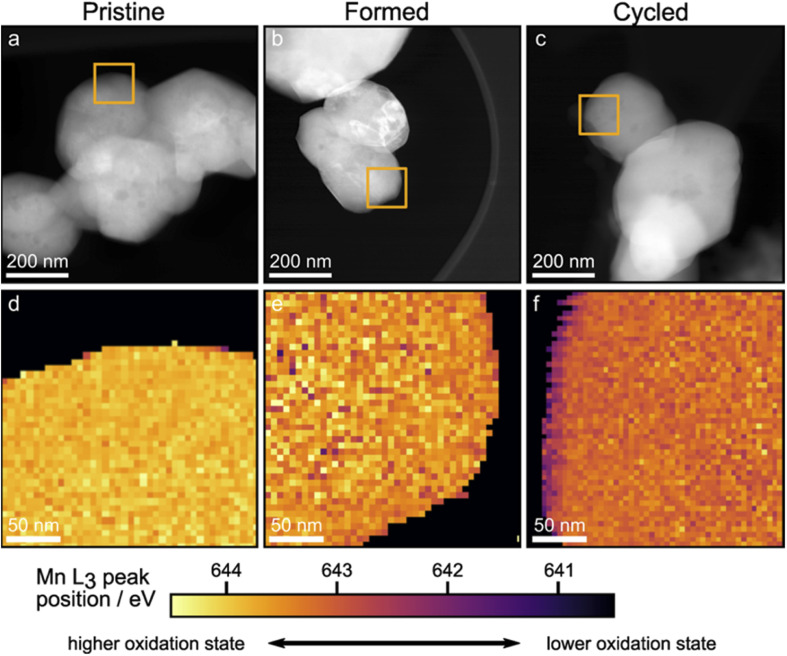
EELS mapping of the Mn *L*_3_ peak position. (a–c), STEM-HAADF images of the *Pristine*, *Formed* and *Cycled*; orange squares outline the region where EELS mapping was conducted. (d–f), EELS mapping of the Mn *L*_3_ peak positions of *Pristine*, *Formed* and *Cycled* respectively. Colour schemes reflect the binding energy from low (lower oxidation state, dark purple) to high (higher oxidation state, yellow).

The STEM-EELS mapping of the Mn *L*_3_ peak positions was carried out to differentiate the binding energy (BE) of the bulk from the surface, hence shedding light on the spatial distribution of different Mn oxidation states. First, suitable particles were identified using high angle annular dark field imaging in STEM mode (HAADF-STEM). Images of the particles are shown in Fig. 6a–c. Orange squares show regions that were mapped using STEM-EELS and the resulting maps of the Mn *L*_3_ peak positions are shown in Fig. 6d–f. The Mn *L*_3_ peak positions for the *Pristine* sample shown in Fig. 6d are uniform throughout the scan area. This is no longer the case after the formation cycles (Fig. 6e): the bulk oxidation state of Mn is reduced, in line with the XANES results. After 100 cycles, the bulk Mn oxidation state is slightly more reduced compared to the *Formed* sample, and a striking reduction of the surface is observed uniformly on the particle surface. The STEM-EELS mapping confirms the XANES findings of lower oxidation state of Mn in the bulk of particles. The surface, however, is revealed to be reduced to a larger degree. Using the spectrum images shown in Fig. 6d–f, line scan profiles across particle surfaces were extracted as described in the Experimental section. The resulting Mn *L*_3_ peak position profiles for the *Pristine*, *Formed* and *Cycled* samples are shown in Fig. S9.† The cascade plots of corresponding spectra are presented in Fig. S10.† The line scan profiles confirm changes of Mn *L*_3_ peak position in the bulk (>15 nm away from the surface) as well as a clear ∼15 nm thick reduced layer on the *Cycled* samples. The reduced surface and sub-surface layer is consistent with Li loss, O loss and densification of the structure.^47^

### 3.4 Transport measurements

**Fig. 7:**
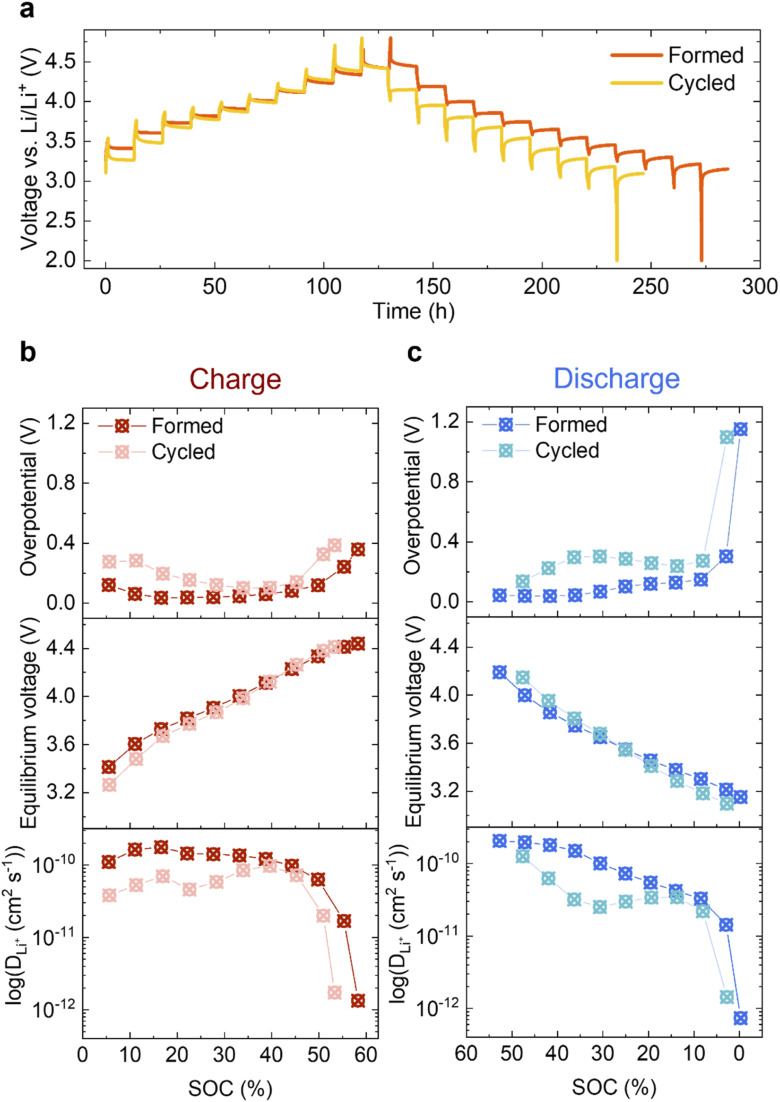
Electrochemical GITT analysis. (a) GITT of the *Formed* and *Cycled*. Charge/discharge pulses applied at C/10 for 1 h, followed by a 12 h relaxation period to reach quasi-equilibrium. Overpotential as a function of SOC for charge (b, top) and discharge (c, top) and equilibrium voltage as a function of SOC for charge (b, middle) and discharge (c, middle). Apparent Li^+^ diffusion coefficient in log scale of charge (b, bottom) and discharge (c, bottom). Here, electrode geometric area is used in the calculation of the diffusion coefficient for comparison purposes (refer to main text for detailed discussions).

Given the limited changes in the bulk material, the increased impedance due to the reduced surface could be a main contributor to the voltage fade observed in Fig. 2c. To study this further, GITT was carried out on the *Formed* and *Cycled* cells as shown in Fig. 7a (magnifications of five different voltage regions are shown in S11†). A clear voltage hysteresis, similar to that seen in a previous study of another Li- and Mn-rich layered oxide is observed.^27^ The electrochemical overpotential is defined as the voltage difference between the potential at the end of a current pulse and the equilibrium potential after relaxing for 12 h (see Fig. S12†). The resulting overpotentials as a function of SOC are plotted in Fig. 7b (top, charge) and Fig. 7c (top, discharge). In both charge and discharge processes, the overpotential of the *Cycled* is higher than the *Formed* at every SOC, whereas the equilibrium potentials achieved when given sufficient time for Li^+^ to diffuse into the bulk are much closer (Fig. 7b and c, middle).

To measure the change in the diffusivity of Li ions in Li_1.2_Ni_0.2_Mn_0.6_O_2_, the apparent diffusion coefficient *D*_Li^+^_ is derived from GITT and presented in Fig. 7b and c (bottom) in log scale as a function of SOC (refer to ESI Note 1 for details†). It is also plotted in linear scale in Fig. S13† for direct comparisons.

The electrochemical active surface area *A* is needed when calculating *D*_Li^+^_ (eqn (1) of ESI Note 1†) since it is effectively used to determine the diffusion path length the ions travel during the relaxation time period after the current pulse. It is, however, difficult to determine which value is appropriate – since neither the particle surface area measured by BET, nor surface area of the electrode (includes conductive carbon additive (Super P) and binder (PVDF)) results in an accurate measure of the diffusion length. However, assuming that the active surface area does not change dramatically during cycling, we can choose a measure of the surface area to study relative changes in the apparent diffusion coefficient over long-term cycling. Here, the geometric area of the circular electrode disc is used (*A* = π*d*^2^/4, 1.131 cm^2^), which is an under-estimation of the real surface area. Li^+^ mobility after extended cycling is decreased at every SOC in both the charge and discharge processes, which is concomitant with the observation that the overpotential has increased. On discharge, the most pronounced decrease in Li mobility and increase in overpotential occur at around 30% SOC, where the largest voltage drop is seen.

## 4. Discussion

### 4.1 Structural evolutions

As mentioned earlier, it is well established that a more condensed layer of spinel/spinel-like/rocksalt forms on the particle surfaces as a consequence of electrochemical cycling, but the degree to which these phases propagate and/or are formed in the bulk remains controversial. In our work, despite using nano-sized Li_1.2_Ni_0.2_Mn_0.6_O_2_ particles (100–500 nm) with a relatively high specific surface area, the quantity of any potentially-formed spinel or rocksalt phase is not sufficient to be picked up by the bulk characterisation techniques of SXRD, ssNMR and Raman spectroscopy: in an ideal layered Li_1.2_Ni_0.2_Mn_0.6_O_2_ structure, octahedrally coordinated Li and TM layers are arranged alternatively in the *c*-axis direction with excess Li accommodated in the TM layers. Transitioning from such layered structure to a standard cubic spinel would necessitate the presence of TM ions in the octahedral sites of both the Li and TM layers, and at some states of charge, Li occupying tetrahedral sites of Li layers. A typical energy-favoured migration path to achieve this is described as follows: Li vacancies are generated during the charging process, allowing TM ions to migrate to the tetrahedral sites in the adjacent Li layers, the TM ions then moving to the octahedral sites in the same layers.^48,49^ Therefore, if significant amounts of TMs in the tetrahedral and octahedral sites of the Li layers are captured by a bulk sensitive technique such as SXRD, this should provide strong evidence that a spinel-like phase is being formed. Fig. 3d shows that the (104) to (108)/(110) peak area ratio, which has been shown by Csernica *et al.* to be a measure of the TMs in the tetrahedral sites,^23^ only changes by 1.27% when comparing the *Pristine* to the *Cycled* samples, suggesting that the concentration of TMs in the tetrahedral sites at the discharged state even after 100 cycles is too low to be detected and that either no significant TM migration is happening or that it is highly transient, because both Mn^4+^ and Mn^3+^ are unstable in tetrahedral coordination,^50,51^ only lower (Mn^2+^) or higher (*e.g.*, Mn^7+^) being stable in this site. In a Rietveld refinement, attempts to place Ni in tetrahedral sites yielded negative site occupancies with close-to-zero absolute values, suggesting that no electron density is present in these sites in discharged samples.

Kleiner *et al.* carried out operando SXRD measurements that monitored the entire charge/discharge process over long-term cycling and captured electron density around tetrahedral sites of Li layers in charged samples using difference Fourier maps, which they attribute to the presence of TM in these sites.^24^ Dogan *et al.* also observed a minor peak at ∼1550 ppm of charged samples (3.6 V) in ^6^Li MAS ssNMR spectra which they ascribed to Li in a LiMn_6_–Mn_tet_ environment.^38^ Based on Rietveld refinements of SXRD patterns, we find the overall quantity of Li/Ni anti-site mixing has not increased after cycling (Fig. 3d), which is in line with the previous reports on similar layered cathode materials with even higher Ni content.^52,53^ As neither occupancy of TM in tetrahedral sites nor octahedral sites of Li layers is increasing noticeably, we conclude that in our samples, continued electrochemical cycling does not lead to the formation of a spinel or spinel-like phase at the bulk level.

### 4.2 Overpotential, voltage fade and Li mobility

Significant differences in the nature of the changes in overpotential and equilibrium voltage with cycling were captured in the GITT measurements (Fig. 7). The latter reflects equilibrium thermodynamic properties: the XANES analysis indicates that bulk Mn is reduced over repetitive cycling (although to a minor extent), which shifts the Fermi level up, leading to a slightly lower OCV, as observed here in the discharged state and in previous studies.^43,54^ Although a difference of approx. 0.15 V in equilibrium voltage is measured at low SOC, there is no substantial difference above ∼15% SOC. Since the overpotential and equilibrium voltage determine the voltage fade,^54^ this indicates that the overpotential is a significant contributor to voltage fade. This is also reflected in the discharge data which shows a large difference in overpotential compared to changes in equilibrium voltage before and after cycling. In a GITT experiment, a small current pulse lithiates a small shell around the surface of a particle. The lithium ions then diffuse into the bulk as equilibrium is established, allowing the (relative) diffusion coefficients, *D*_Li^+^_ to be estimated – or at least compared. The overpotential is strongly influenced by the Li transport through the surface layers, while the Li^+^ diffusion coefficients are affected by transport through both the surface and bulk layers. A high apparent diffusion coefficient *D*_Li^+^_ and low overpotential (for the *Formed* sample) are observed at the beginning of charge (Fig. 7b). This is because the cathode material is fully lithiated initially, rendering Li on the surface readily removable;^55^ transport through the bulk is also moderately fast initially. Note that this is in contrast to stoichiometric NMCs, which show very low values of *D*_Li^+^_ at SOC below approximately 15% due to the low vacancy concentrations.^56^ Although direct comparisons are difficult, potential reasons for this are (i) the higher overall values of *D*_Li^+^_ for NMCs, which unlike Li-rich materials become much faster at intermediate SOCs, (ii) the Li-rich materials are presumably less ordered at 100% SOC with more intrinsic vacancies and defects. The values of *D*_Li^+^_ decreased with cycling, although *D*_Li^+^_ dropped rapidly both in the *Formed* and *Cycled* samples above approximately 50% SOC. There seem to be two regions where higher overpotentials are observed (i) at low-intermediate SOCs, where they increase with cycling, and (ii) at high SOC, where they remain very high, both before and after long term cycling. The latter overpotential is most likely associated with the processes involved in anion redox, which may also involve migration of ions. The former increase tracks (somewhat) with decreases in *D*_Li^+^_, suggesting that transport through the surface and sub-surface regions impedes delithiation. The STEM-EELS mapping suggests that can be related to the Mn redox processes largely involving more reduced regions near or close to the surface. The very low *D*_Li^+^_ values at the end of charge were instantly restored when the direction of applied current was reversed at the beginning of discharge, which is frequently seen in the anion redox chemistry.^57,58^ The diffusivities gradually reduce as the SOC decreases (Fig. 7c), the drop becomes pronounced after extended cycling. This drop clearly correlates with the increase in overpotential, whilst the equilibrium potential remains largely unchanged. It again strongly suggests that this is closely linked to surface-specific electrochemical processes. The overpotential and drop in *D*_Li^+^_ is larger on lithiation, contributing to the hysteresis seen at lower SOCs on extended cycling. Thus, we suggest that this is related to the transport through the surface and sub-surface reduced (densified) layers which, will strongly depend on SOC (and thus the oxidation state of Mn). While some reduction of Mn in the bulk may occur upon extended cycling it does not appear to be sufficient to account for the large capacity seen at low voltages because approximately 35% of the capacity is obtained below 3.2 V even in the slow C/10 recovery cycles performed before the GITT experiment.

## 5. Conclusions

The voltage decay has long been considered as one of the most prominent degradation problems of Li- and Mn-rich layered cathode materials, hindering their use on a larger scale. In contrast to a widely accepted theory that such voltage decay results from a layered-to-spinel phase change in the bulk, we propose that the severely reduced particle surface, instead, plays the key role. This conclusion is based on an array of characterisation methods, especially the STEM-EELS mapping and GITT studies that captured the surface-specific material property changes and electrochemical processes. In addition, a detailed analysis of samples harvested at different stages during the aging process, with tools that are sensitive to both long-range and short-range order, showed that in contrast to the substantial structural changes that occurred in the formation cycles, further evolution of the bulk structure became much less rapid. In particular, no additional phases were detected after extended cycling. ^7^Li NMR shows that, even after 100 cycles at C/2, Li can still be reversibly inserted into both the lithium and transition metal layers. Voltage fade is, however, very clearly present under these conditions. While more aggressive cycling or slow rates may induce more severe degradation and potentially bulk structural changes, this work shows that voltage fade is still seen when there is little or no bulk structural change. Finally, this study indicates that approaches to stabilise the surface and sub-surface regions (*e.g.* by coatings or surface-specific dopings or the use of different electrolytes) may provide an effective route forward to reduce voltage drop and maintain high energy density over long-term cycling, thus helping to make Li- and Mn-rich layered oxides commercially viable.

## Conflicts of interest

There are no conflicts to declare.

## Supplementary Material

TA-010-D2TA04876K-s001

## References

[cit1] Goodenough J. B., Kim Y. (2010). Chem. Mater..

[cit2] Armand M., Tarascon J. M. (2008). Nature.

[cit3] Jung R., Metzger M., Maglia F., Stinner C., Gasteiger H. A. (2017). J. Electrochem. Soc..

[cit4] Hy S., Liu H., Zhang M., Qian D., Hwang B. J., Meng Y. S. (2016). Energy Environ. Sci..

[cit5] Li W., Song B., Manthiram A. (2017). Chem. Soc. Rev..

[cit6] Zheng J., Myeong S., Cho W., Yan P., Xiao J., Wang C., Cho J., Zhang J. G. (2017). Adv. Energy Mater..

[cit7] Rozier P., Tarascon J. M. (2015). J. Electrochem. Soc..

[cit8] Hong J., Gwon H., Jung S.-K., Ku K., Kang K. (2015). J. Electrochem. Soc..

[cit9] Sun S., Li J., Xu C., Zhai T., Xia H. (2022). J. Mater. Chem. A.

[cit10] Bréger J., Jiang M., Dupré N., Meng Y. S., Shao-Horn Y., Ceder G., Grey C. P. (2005). J. Solid State Chem..

[cit11] Kalyani P., Chitra S., Mohan T., Gopukumar S. (1999). J. Power Sources.

[cit12] Yabuuchi N., Yoshii K., Myung S. T., Nakai I., Komaba S. (2011). J. Am. Chem. Soc..

[cit13] Muhammad S., Kim H., Kim Y., Kim D., Song J. H., Yoon J., Park J. H., Ahn S. J., Kang S. H., Thackeray M. M., Yoon W. S. (2016). Nano Energy.

[cit14] Thackeray M. M., Kang S. H., Johnson C. S., Vaughey J. T., Benedek R., Hackney S. A. (2007). J. Mater. Chem..

[cit15] Yu D. Y. W., Yanagida K., Kato Y., Nakamura H. (2009). J. Electrochem. Soc..

[cit16] Assat G., Tarascon J. M. (2018). Nat. Energy.

[cit17] Liu H., Harris K. J., Jiang M., Wu Y., Goward G. R., Botton G. A. (2018). ACS Nano.

[cit18] Gu M., Belharouak I., Zheng J., Wu H., Xiao J., Genc A., Amine K., Thevuthasan S., Baer D. R., Zhang J. G., Browning N. D., Liu J., Wang C. (2013). ACS Nano.

[cit19] Mohanty D., Sefat A. S., Li J., Meisner R. A., Rondinone A. J., Payzant E. A., Abraham D. P., Wood D. L., Daniel C. (2013). Phys. Chem. Chem. Phys..

[cit20] Boulineau A., Simonin L., Colin J. F., Bourbon C., Patoux S. (2013). Nano Lett..

[cit21] Gu M., Belharouak I., Zheng J., Wu H., Xiao J., Genc A., Amine K., Thevuthasan S., Baer D. R., Zhang J. G., Browning N. D., Liu J., Wang C. (2013). ACS Nano.

[cit22] Yan P., Zheng J., Tang Z. K., Devaraj A., Chen G., Amine K., Zhang J. G., Liu L. M., Wang C. (2019). Nat. Nanotechnol..

[cit23] Csernica P. M., Kalirai S. S., Gent W. E., Lim K., Yu Y. S., Liu Y., Ahn S. J., Kaeli E., Xu X., Stone K. H., Marshall A. F., Sinclair R., Shapiro D. A., Toney M. F., Chueh W. C. (2021). Nat. Energy.

[cit24] Kleiner K., Strehle B., Baker A. R., Day S. J., Tang C. C., Buchberger I., Chesneau F. F., Gasteiger H. A., Piana M. (2018). Chem. Mater..

[cit25] Teufl T., Strehle B., Müller P., Gasteiger H. A., Mendez M. A. (2018). J. Electrochem. Soc..

[cit26] Strehle B., Kleiner K., Jung R., Chesneau F., Mendez M., Gasteiger H. A., Piana M. (2017). J. Electrochem. Soc..

[cit27] Yu Z., Ning F., Shang H., Song J., Yao T., Sun Z., Chu W., Xia D. (2021). J. Phys. Chem. C.

[cit28] Zhang M., Kitchaev D. A., Lebens-Higgins Z., Vinckeviciute J., Zuba M., Reeves P. J., Grey C. P., Whittingham M. S., Piper L. F. J., Van der Ven A., Meng Y. S. (2022). Nat. Rev. Mater..

[cit29] Gu M., Belharouak I., Genc A., Wang Z., Wang D., Amine K., Gao F., Zhou G., Thevuthasan S., Baer D. R., Zhang J. G., Browning N. D., Liu J., Wang C. (2012). Nano Lett..

[cit30] Jarvis K., Wang C. C., Varela M., Unocic R. R., Manthiram A., Ferreira P. J. (2017). Chem. Mater..

[cit31] Zheng J., Gu M., Genc A., Xiao J., Xu P., Chen X., Zhu Z., Zhao W., Pullan L., Wang C., Zhang J. G. (2014). Nano Lett..

[cit32] Dose W. M., Temprano I., Allen J. P., Björklund E., O’Keefe C. A., Li W., Mehdi B. L., Weatherup R. S., De Volder M. F. L., Grey C. P. (2022). ACS Appl. Mater. Interfaces.

[cit33] Xu J., Sun M., Qiao R., Renfrew S. E., Ma L., Wu T., Hwang S., Nordlund D., Su D., Amine K., Lu J., McCloskey B. D., Yang W., Tong W. (2018). Nat. Commun..

[cit34] de la Peña F. , Prestat E., Fauske V. T., Burdet P., Furnival T., Jokubauskas P., Lähnemann J., Nord M., Ostasevicius T., MacArthur K. E., Johnstone D. N., Sarahan M., Taillon J., Aarholt T., Migunov V., Eljarrat A., Caron J., Poon T., Mazzucco S., Martineau B., Somnath S., Slater T., Francis C., Tappy N., Walls M., Cautaerts N., Winkler F. and Donval G., hyperspy/hyperspy, Release v1.6.4, 2021, DOI: 10.5281/zenodo.5082777

[cit35] Strobel P., Lambert-Andron B. (1988). J. Solid State Chem..

[cit36] Massarotti V., Bini M., Capsoni D., Altomare A., Moliterni A. G. G. (1997). J. Appl. Crystallogr..

[cit37] Jarvis K. A., Wang C. C., Manthiram A., Ferreira P. J. (2014). J. Mater. Chem. A.

[cit38] Dogan F., Long B. R., Croy J. R., Gallagher K. G., Iddir H., Russell J. T., Balasubramanian M., Key B. (2015). J. Am. Chem. Soc..

[cit39] Shimoda K., Yazawa K., Matsunaga T., Murakami M., Yamanaka K., Ohta T., Matsubara E., Ogumi Z., Abe T. (2020). Sci. Rep..

[cit40] Hong J., Seo D. H., Kim S. W., Gwon H., Oh S. T., Kang K. (2010). J. Mater. Chem..

[cit41] Huang J. X., Li B., Liu B., Liu B. J., Zhao J. B., Ren B. (2016). J. Power Sources.

[cit42] Lanz P., Villevieille C., Novák P. (2014). Electrochim. Acta.

[cit43] Hu E., Yu X., Lin R., Bi X., Lu J., Bak S., Nam K. W., Xin H. L., Jaye C., Fischer D. A., Amine K., Yang X. Q. (2018). Nat. Energy.

[cit44] Croy J. R., Iddir H., Gallagher K., Johnson C. S., Benedek R., Balasubramanian M. (2015). Phys. Chem. Chem. Phys..

[cit45] Yoon W. S., Balasubramanian M., Chung K. Y., Yang X. Q., McBreen J., Grey C. P., Fischer D. A. (2005). J. Am. Chem. Soc..

[cit46] Dau H., Liebisch P., Haumann M. (2003). Anal. Bioanal. Chem..

[cit47] Xu B., Fell C. R., Chi M., Meng Y. S. (2011). Energy Environ. Sci..

[cit48] Croy J. R., Gallagher K. G., Balasubramanian M., Long B. R., Thackeray M. M. (2014). J. Electrochem. Soc..

[cit49] Mohanty D., Li J., Abraham D. P., Huq A., Payzant E. A., Wood D. L., Daniel C. (2014). Chem. Mater..

[cit50] Gallagher K. G., Croy J. R., Balasubramanian M., Bettge M., Abraham D. P., Burrell A. K., Thackeray M. M. (2013). Electrochem. Commun..

[cit51] Reed J., Ceder G. (2004). Chem. Rev..

[cit52] Xu C., Märker K., Lee J., Mahadevegowda A., Reeves P. J., Day S. J., Groh M. F., Emge S. P., Ducati C., Mehdi B. L., Tang C. C., Grey C. P. (2021). Nat. Mater..

[cit53] Friedrich F., Strehle B., Freiberg A. T. S., Kleiner K., Day S. J., Erk C., Piana M., Gasteiger H. A. (2019). J. Electrochem. Soc..

[cit54] Zhu Z., Yu D., Yang Y., Su C., Huang Y., Dong Y., Waluyo I., Wang B., Hunt A., Yao X., Lee J. (2019). Nat. Energy.

[cit55] Zheng J., Shi W., Gu M., Xiao J., Zuo P., Wang C., Zhang J.-G. (2013). J. Electrochem. Soc..

[cit56] Grenier A., Reeves P. J., Liu H., Seymour I. D., Märker K., Wiaderek K. M., Chupas P. J., Grey C. P., Chapman K. W. (2020). J. Am. Chem. Soc..

[cit57] Yu H., Wang Y., Asakura D., Hosono E., Zhang T., Zhou H. (2012). RSC Adv..

[cit58] Liu J., Chen H., Xie J., Sun Z., Wu N., Wu B. (2014). J. Power Sources.

