# Supplementary material for: Surface reduction in lithium- and manganese-rich layered cathodes for lithium ion batteries drives voltage decay
Source: J Mater Chem A Mater. 2022 Sep 28;10(41):21941–54. doi: 10.1039/d2ta04876k (PMC9595402; doi:10.1039/d2ta04876k)
Supplement: TA-010-D2TA04876K-s001 [file TA-010-D2TA04876K-s001.pdf]

Electronic Supplementary Material (ESI) for Journal of Materials Chemistry A.
This journal is © The Royal Society of Chemistry 2022

Supplementary Information

Surface reduction in lithium- and manganese-rich layered cathodes for lithium ion batteries drives voltage decay

Bo Wen,[a,c] Farheen N. Sayed,[b,e] Wesley M. Dose,[a,b,e] Jędrzej K. Morzy,[a,d,e] Yeonguk Son,[a,f] Supreeth Nagendran,[b] Caterina Ducati,[d,e] Clare P. Grey*[b,e] and Michael F. L. De Volder*[a,e]

[a]*Department of Engineering, University of Cambridge, 17 Charles Babbage Road, Cambridge, CB3 0FS, UK. E-mail: mfld2@cam.ac.uk*

[b]*Yusuf Hamied Department of Chemistry, University of Cambridge, Lensfield Road, Cambridge, CB2 1EW, UK. E-mail: cpg27@cam.ac.uk*

[c]*Cambridge Graphene Centre, University of Cambridge, 9 JJ Thomson Avenue, Cambridge, CB3 0FA, UK.*

[d]*Department of Materials Science and Metallurgy, University of Cambridge, 27 Charles Babbage Road, Cambridge, CB3 0FS, UK.*

[e]*The Faraday Institution, Quad One, Harwell Science and Innovation Campus, Didcot, OX11 0RA, UK.*

[f]*Department of Chemical Engineering, Changwon National University, Gyeongsangnam-do, 51140, Republic of Korea*

Table S1: Rietveld refinement parameters obtained for $Li_{1.2}Ni_{0.2}Mn_{0.6}O_2$ from HT, MHT and BM

**Sample**	**a, b (Å)**	**c (Å)**	**V (Å^3)**	**c/a**	**R_{wp} (%)**	**GoF**
HT	2.8598(1)	14.2570(7)	100.976(8)	4.985	4.63	1.22
MHT	2.8581(1)	14.2557(9)	100.848(11)	4.988	4.56	1.17
BM	2.8615(1)	14.2538(6)	101.073(6)	4.981	4.75	1.23

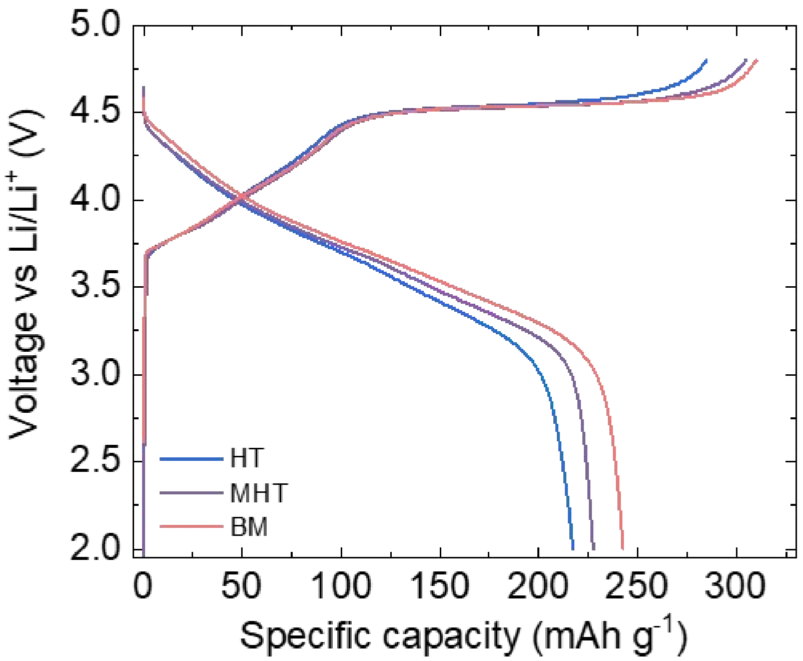

Figure S1. The first electrochemical formation cycle of $Li_{1.2}Ni_{0.2}Mn_{0.6}O_2$ synthesised via three approaches (current density=20 mA g^{-1}). The first discharge specific capacities obtained this way were used for determining current densities of C-rates for long-term cycling and GITT.

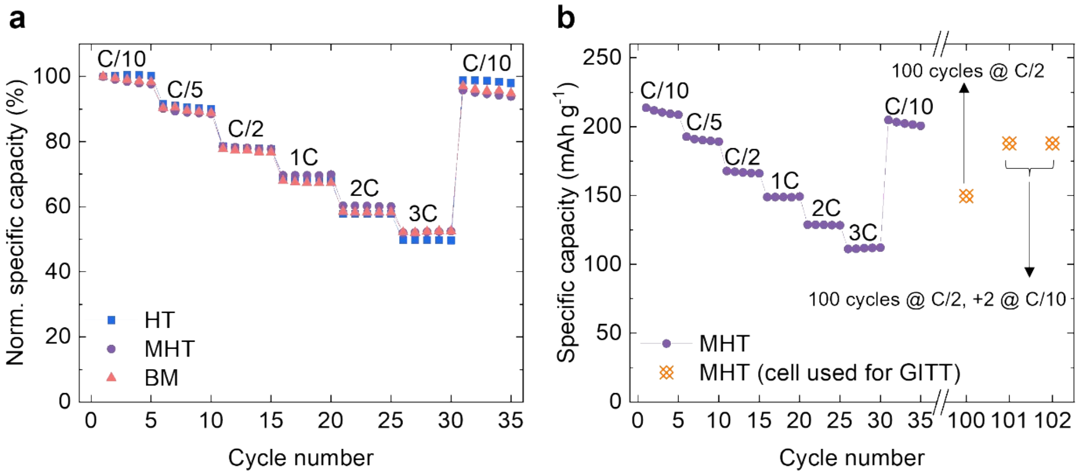

Figure S2. (a) Normalised plot of rate performance of $Li_{1.2}Ni_{0.2}Mn_{0.6}O_2$ synthesised via different routes at various C-rates. (b) The specific capacities of an MHT cell after being cycled at C/2 for 100 cycles and then cycled at C/10 for 2 cycles (orange crosses). This cell was later used for GITT test (annotated as Cycled in Fig. 7). The MHT cell used in (a) is also plotted for comparison (purple). The areal loadings of the active material in all cells were carefully controlled to ~3.7 mg cm^{-2} for a fair comparison.

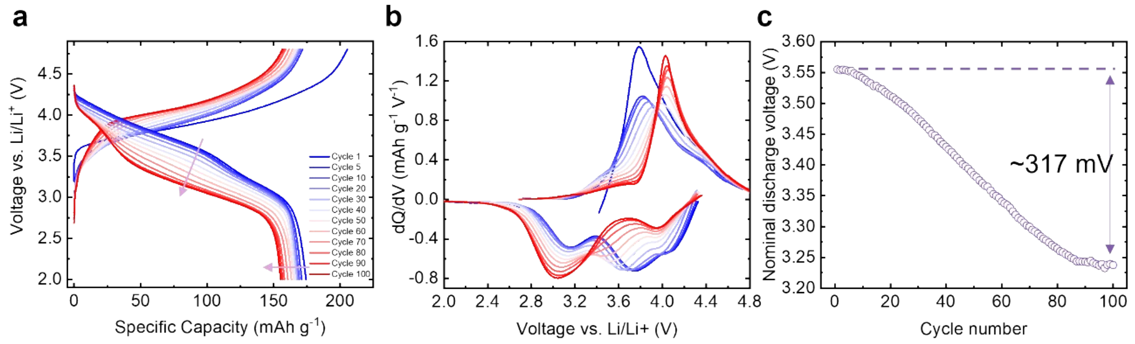

Figure S3. Electrochemical characterisations of $Li_{1.2}Ni_{0.2}Mn_{0.6}O_2$ from MHT approach. (a) Galvanostatic cycling of MHT for 100 cycles at C/2 in a voltage window of 4.8-2 V. Purple arrows indicate evolutions of discharge voltage curves. Cycle 1 is the first cycle after three formation cycles at 20 mA g^{-1}. (b) Evolution of dQ dV^{-1} plot for 100 cycles derived from (a). (c) Nominal discharge voltage over long-term cycling.

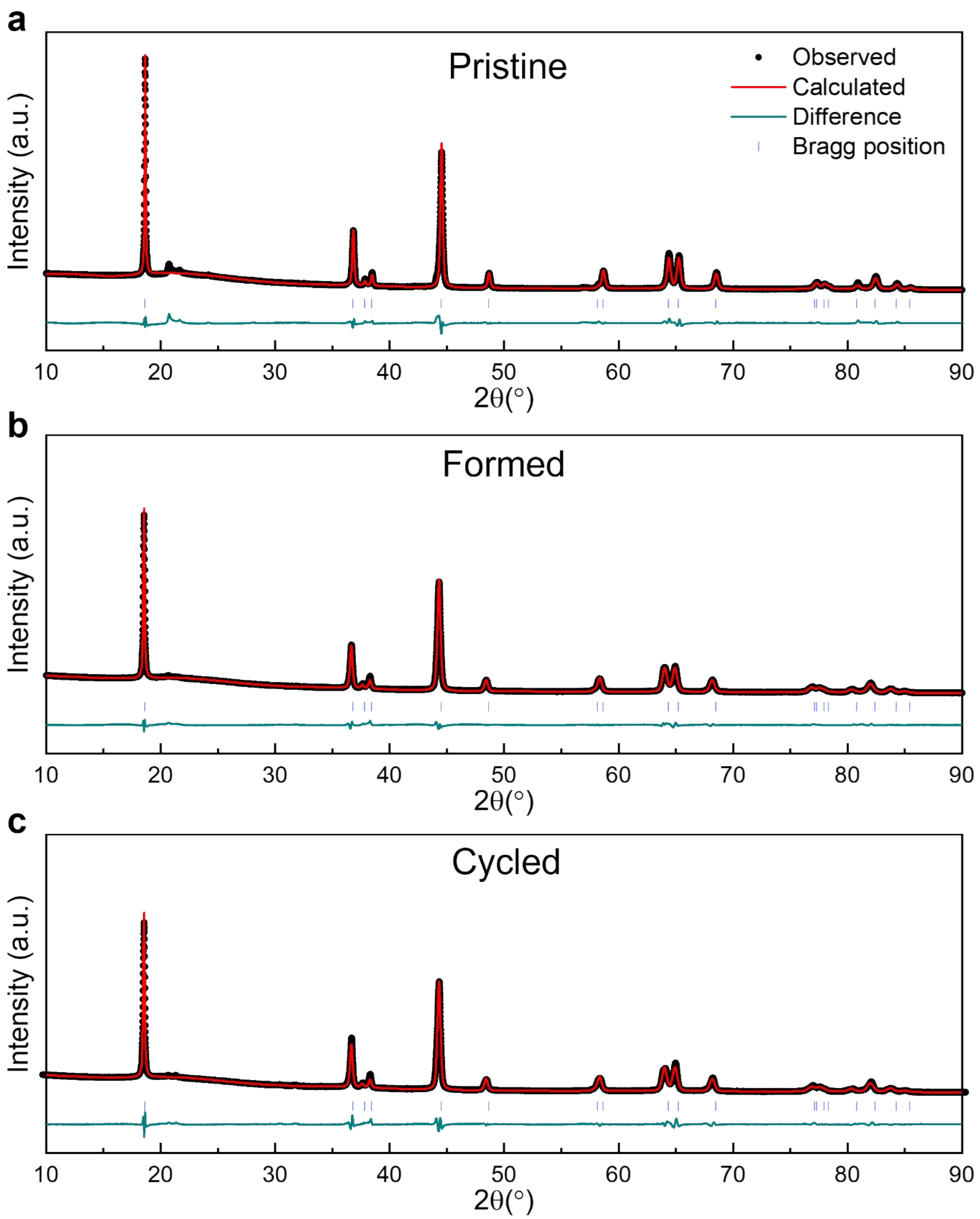

Figure S4. Rietveld refinement of SXRD of (a) *Pristine*; (b) *Formed* and (c) *Cycled* samples. X-ray wavelength converted to Cu Kα. The refinement was based on $R\bar{3}m$ space group.

Table S2: Rietveld refinement results for *Pristine*

**Wyckoff positions**	**Atom**	**x**	**y**	**z**	**Occupancy**
3a	Li	0	0	0	0.978(1)
	Ni	0	0	0	0.022(1)
3b	Li	0	0	0.5	0.222(1)
	Ni	0	0	0.5	0.178(1)
	Mn	0	0	0.5	0.6
6c	O	0	0	0.24189(7)	1

Space group: $R\bar{3}m$

Rwp=5.14%, GoF=12.54, c/a=4.987

Table S3: Rietveld refinement results for *Formed*

**Wyckoff positions**	**Atom**	**x**	**y**	**z**	**Occupancy**
3a	Li	0	0	0	0.976(1)
	Ni	0	0	0	0.024(1)
3b	Li	0	0	0.5	0.224(1)
	Ni	0	0	0.5	0.176(1)
	Mn	0	0	0.5	0.6
6c	O	0	0	0.24183(4)	1

Space group: $R\bar{3}m$

Rwp=3.28%, GoF=7.90, c/a=4.991

Table S4: Rietveld refinement results for *Cycled*

**Wyckoff positions**	**Atom**	**x**	**y**	**z**	**Occupancy**
3a	Li	0	0	0	0.984(1)
	Ni	0	0	0	0.016(1)
3b	Li	0	0	0.5	0.216(1)
	Ni	0	0	0.5	0.184(1)
	Mn	0	0	0.5	0.6
6c	O	0	0	0.24163(4)	1

Space group: $R\bar{3}m$

Rwp=4.43%, GoF=10.76, c/a=4.994

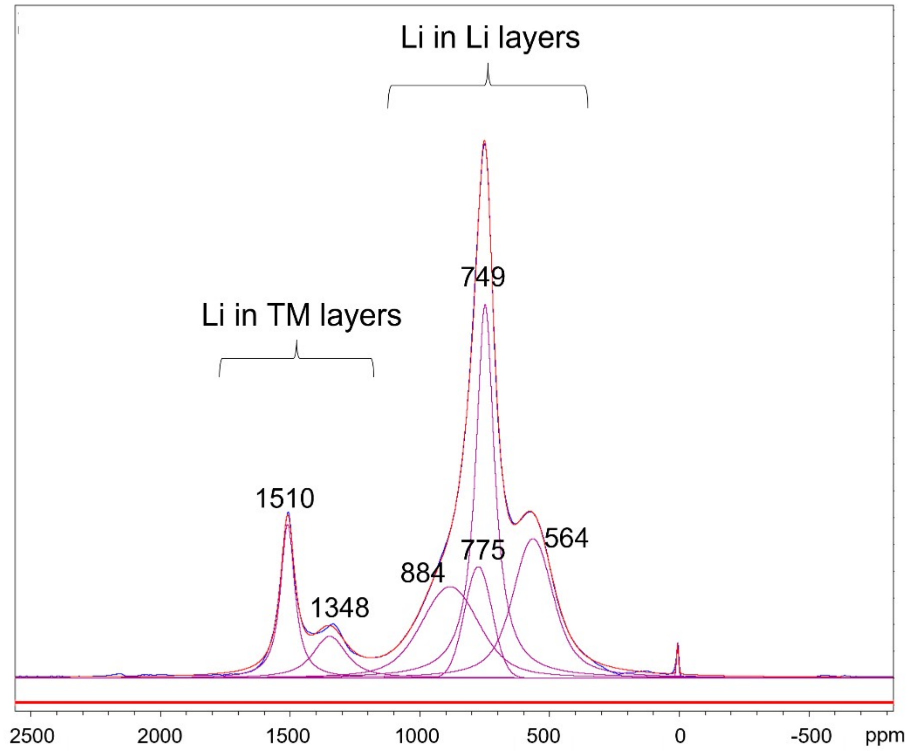

Figure S5. ^{7}Li MAS ssNMR MATPAS spectrum and peak fittings of the *Pristine* sample. The spectrum is de-convoluted (using the inbuilt SOLA package in Topspin) into two main groups of resonances: Li in Li layers and Li in TM layers. Resonances at 749, 775 and 1510 ppm are attributed to Li_2MnO_3-like local structures; the resonance at 1348 ppm is a signature of Li surrounded by 5 Mn^{4+} and 1 Ni^{2+}; the resonances at 564 and 884 ppm are assigned to Li in Li layers with different numbers of TM and Li ions in the first and second coordination shells.[1,2]

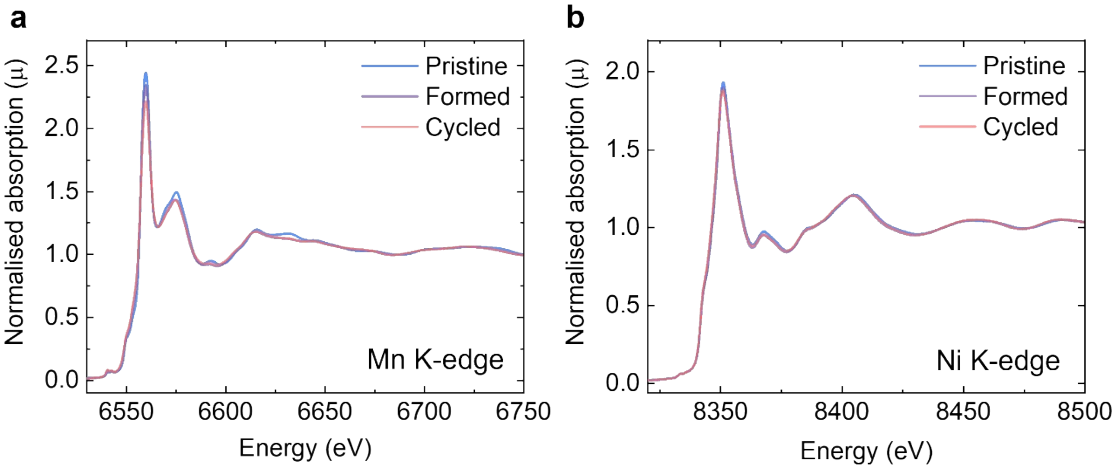

Figure S6. Full XANES spectra of (a) Mn and (b) Ni K-edges (extended Figure 5 in the main text).

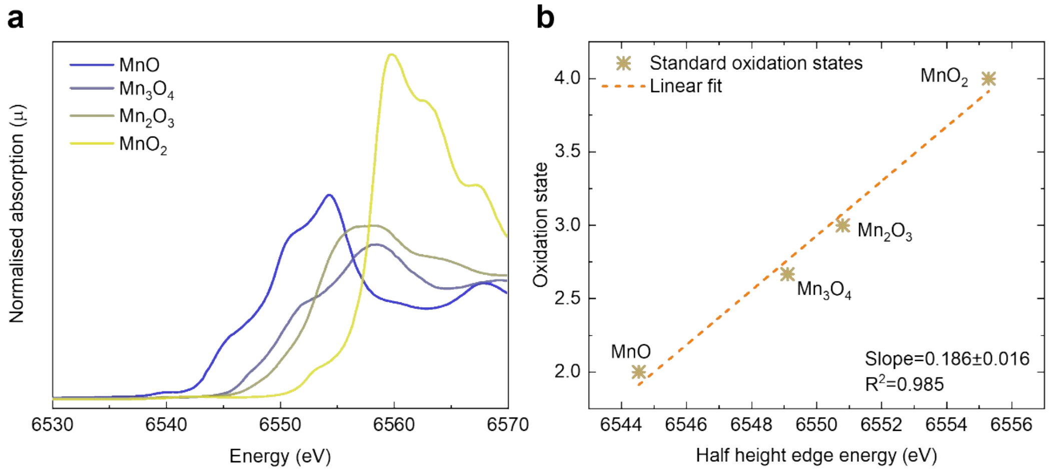

Figure S7. (a) XANES scans of Mn standards. (b) Linear fitting of Mn oxidation state against edge energy. The edge energies (as measured by XAS) of standard Mn-containing samples corresponding to absorption coefficient μ=0.5 (half height) are plotted against their respective oxidation states, yielding a linear fit with R^2=0.985 that is used for determining the oxidation states of the *Pristine*, *Formed* and *Cycled*. The half-height method is adopted from Dau et al.[3]

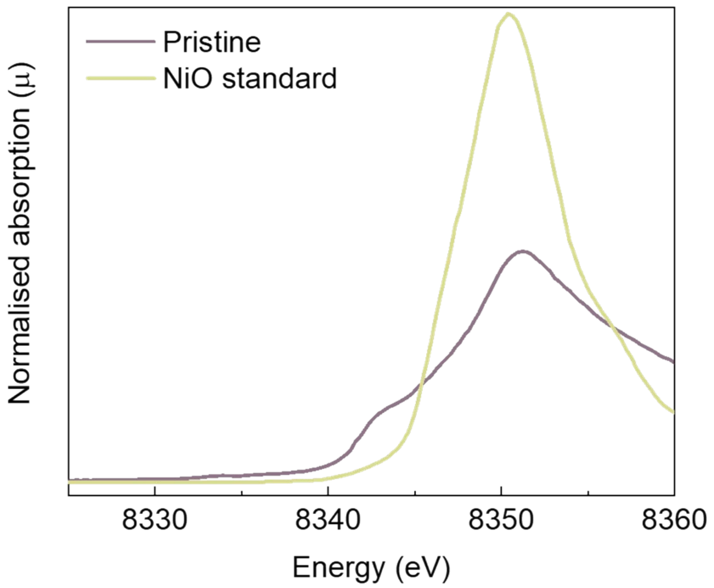

Figure S8. XANES Ni-edge scans of the *Pristine* and NiO standard. The edge position of the *Pristine* corresponds to higher energy compared to the standard, which is an indicator that Ni oxidation state is higher than +2. It correlates well with Mn oxidation state being less than +4 as detailed in the main text.

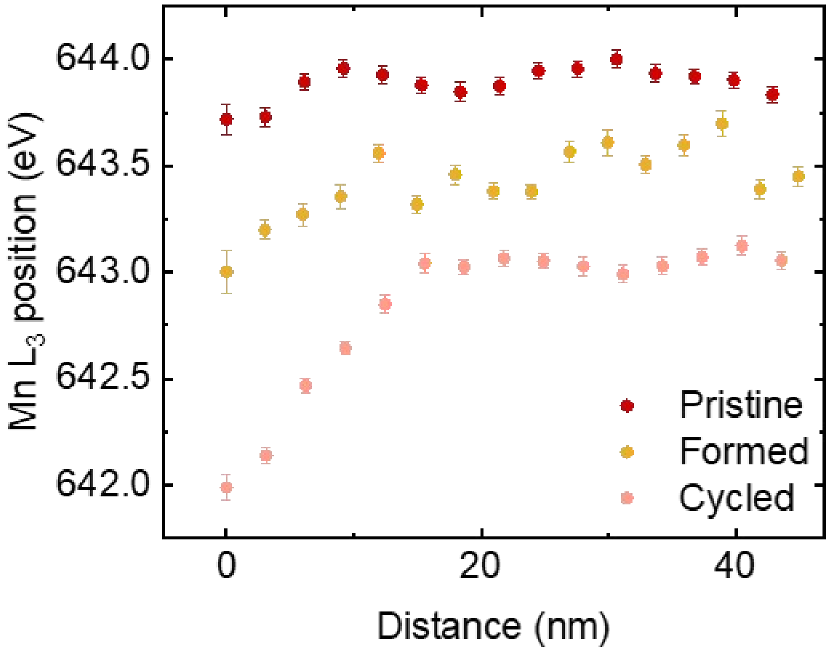

Figure S9. EELS Mn L_3 peak position changes across particle surface. Distance is measured from the edge of the particle, where error bars are standard deviation of fit of the Gaussian component of the model (refer to Experimental section for more details).

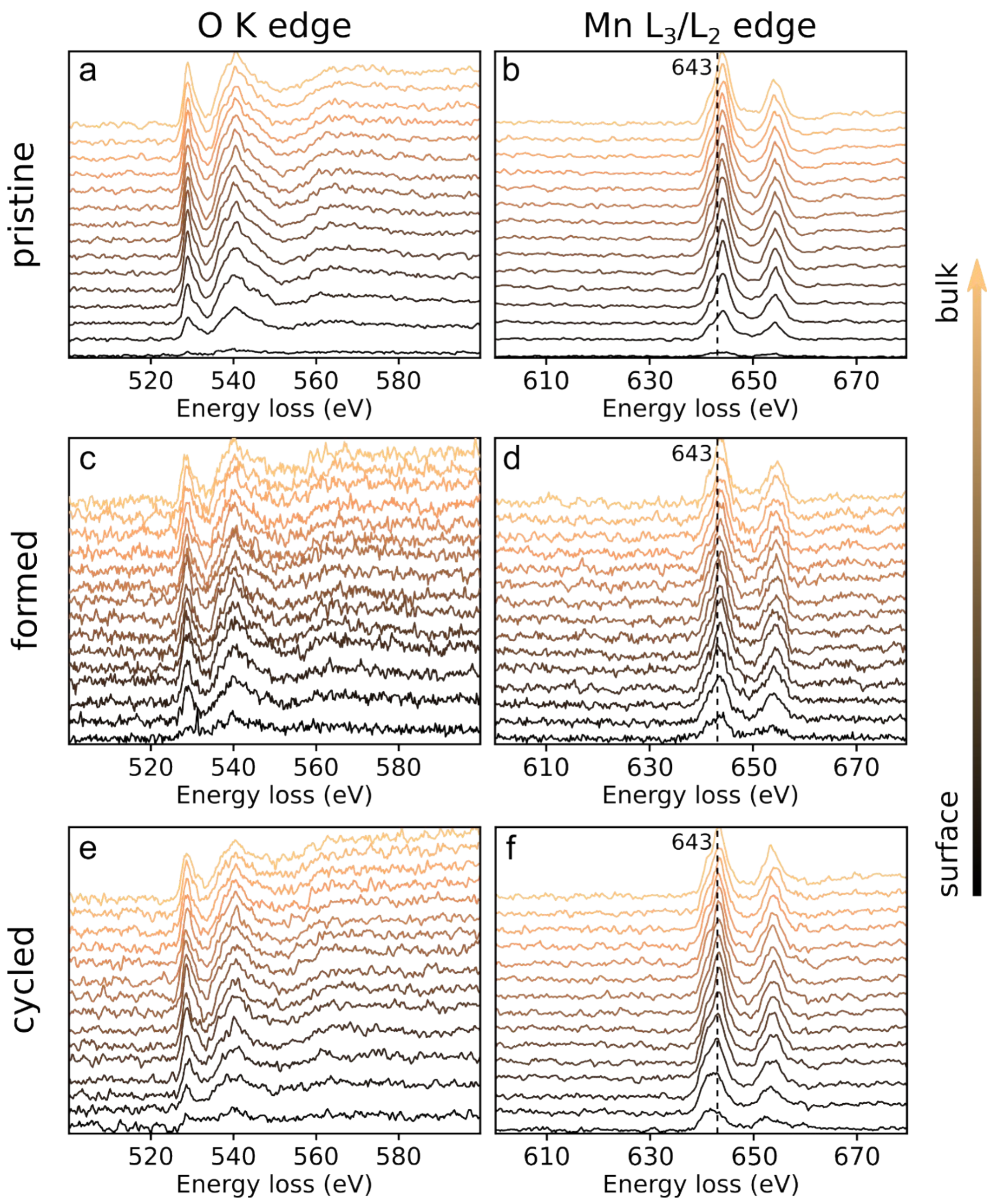

Figure S10. Cascade plots of the EELS O K and Mn L edges corresponding to the line scan Mn L3 peak position shown in Fig. S9. (a-b) *Pristine*, (c-d) *Formed*, and (e-f) *Cycled* samples; left column: O K edge, right column: Mn L edge. The 643 eV energy loss is highlighted with a dashed line in the Mn L edge plots to guide the eye.

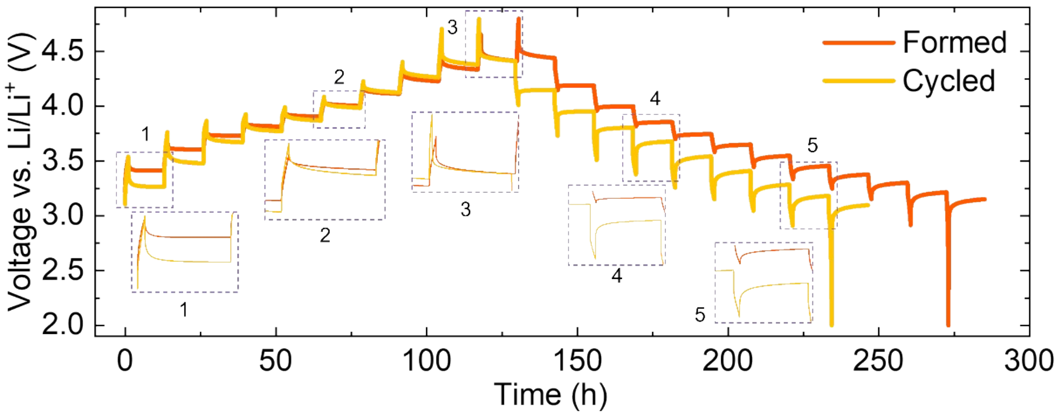

Figure S11 Close-ups of five different voltage regions of the GITT. Quasi-equilibriums are established after 12-hour relaxation.

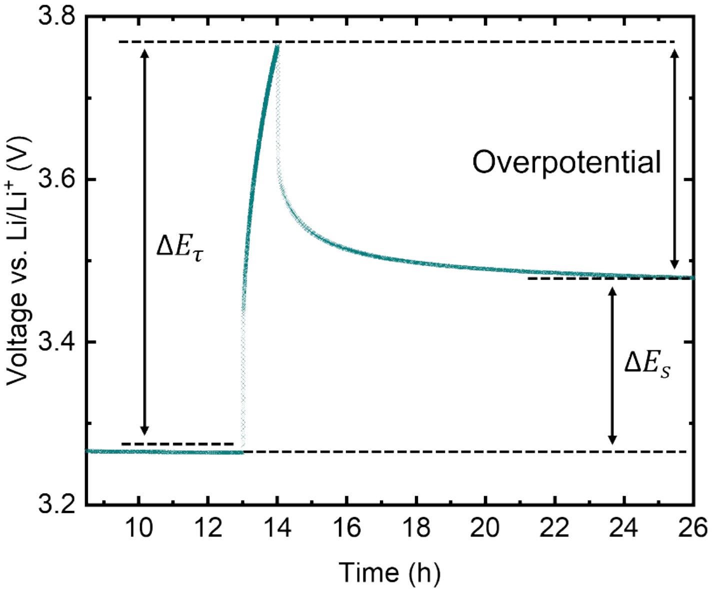

Figure S12. Illustration of overpotential, Δ^{E_τ} and Δ^{E_s}. The overpotential is defined as the voltage difference between the potential at the end of a current pulse and the equilibrium potential after relaxing for 12 h. Δ^{E_τ} is the total transient voltage change of the cell when a constant current is applied for time τ. Δ^{E_s} is the difference between the equilibrium voltages after one current pulse.[4]

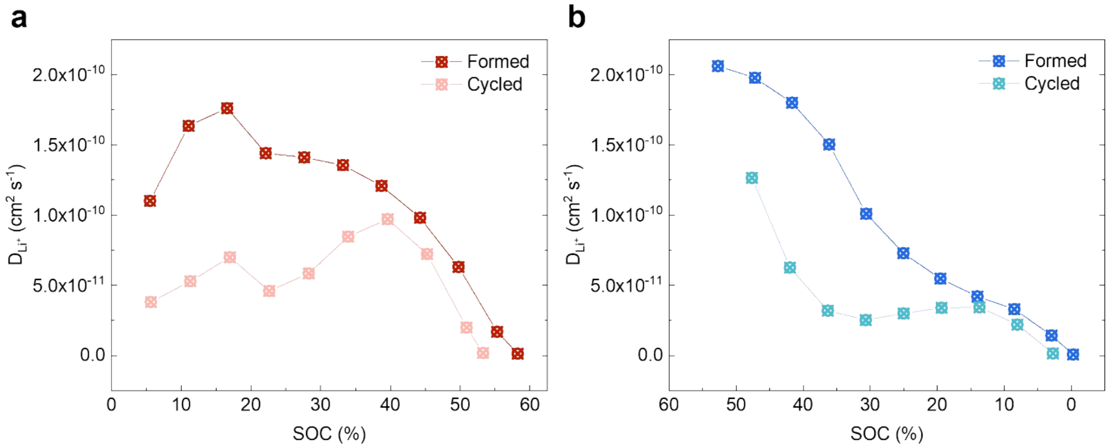

Figure S13. Apparent Li^+ diffusion coefficient in linear scale for (a) charge and (b) discharge, derived from GITT measurements.

Supplementary Note 1:

The apparent diffusion coefficient D_{Li^+} is derived from GITT using the following equation based on Fick's second law of diffusion with a simplification due to the quasi-linear relationship between the voltage during current pulsing E_τ and square root of the current pulse time $\sqrt{\tau}$:[4,5]

$$D_{Li^+} = \frac{4}{\pi\tau}\left(\frac{mV_m}{MA}\right)^2\left(\frac{\Delta E_s}{\Delta E_\tau}\right)^2 \quad (1)$$

where m is the weight of active material, M and V_m are molar mass and molar volume, respectively. ΔE_τ and ΔE_s are the voltage changes during current pulsing and relaxation and were shown schematically in Fig. S12.

Reference

1 M. Jiang, B. Key, Y. S. Meng and C. P. Grey, *Chem. Mater.*, 2009, **21**, 2733–2745.

2 F. Dogan, B. R. Long, J. R. Croy, K. G. Gallagher, H. Iddir, J. T. Russell, M. Balasubramanian and B. Key, *J. Am. Chem. Soc.*, 2015, **137**, 2328–2335.

3 H. Dau, P. Liebisch and M. Haumann, *Anal. Bioanal. Chem.*, 2003, **376**, 562–583.

4 W. Weppner and R. A. Huggins, *J. Electrochem. Soc.*, 1977, **124**, 1569–1578.

5 K. M. Shaju, G. V. Subba Rao and B. V. R. Chowdari, *J. Electrochem. Soc.*, 2003, **150**, A1.
